# Development of a Physical Model-Based Algorithm for the Detection of Single-Nucleotide Substitutions by Using Tiling Microarrays

**DOI:** 10.1371/journal.pone.0054571

**Published:** 2013-01-28

**Authors:** Naoaki Ono, Shingo Suzuki, Chikara Furusawa, Hiroshi Shimizu, Tetsuya Yomo

**Affiliations:** 1 Graduate School of Information Science, Nara Institute of Science and Technology, Ikoma, Nara, Japan; 2 Quantitative Biology Center, RIKEN, Suita, Osaka, Japan; 3 Graduate School of Information Science and Technology, Osaka University, Suita, Osaka, Japan; 4 Graduate School of Frontier Biosciences, Osaka University, Suita, Osaka, Japan; 5 ERATO, JST, Suita, Osaka, Japan; University of Catania, Italy

## Abstract

High-density DNA microarrays are useful tools for analyzing sequence changes in DNA samples. Although microarray analysis provides informative signals from a large number of probes, the analysis and interpretation of these signals have certain inherent limitations, namely, complex dependency of signals on the probe sequences and the existence of false signals arising from non-specific binding between probe and target. In this study, we have developed a novel algorithm to detect the single-base substitutions by using microarray data based on a thermodynamic model of hybridization. We modified the thermodynamic model by introducing a penalty for mismatches that represent the effects of substitutions on hybridization affinity. This penalty results in significantly higher detection accuracy than other methods, indicating that the incorporation of hybridization free energy can improve the analysis of sequence variants by using microarray data.

## Introduction

High-density oligonucleotide microarrays have recently become widely utilized not only for analysis of gene expression, but also for analysis of genomic sequences [1], [2], [3]. For example, microarrays designed for the detection of single-nucleotide polymorphisms (SNPs) have been used in the field of medicine to study genomic sequences and to determine disease susceptibility [4], [5], [6]. Oligonucleotide probes hybridize more efficiently to DNA targets whose sequence is exactly complementary than to targets which have mismatch, i.e., only partially or imperfectly match the probes. Statistical analyses have been developed to detect the difference of probe intensity between two strains. Gresham *et al.*, proposed an algorithm named SNPscanner for high-density tiling microarrays [7]. It models the decrease of intensity caused by a mismatch due to a SNP as a function of probe position and bases reference sequence adjacent to the SNP, and detect SNPs by comparing the observed intensity with prediction of the models to find the best likelihood score.

While recent rapid development in the study of next-generation sequencers has drastically reduced the cost of whole genome sequencing [8], [9], the use of high-density microarrays for genomic analysis has advantages because of simplicity and cost of the experiments [10]. Although microarray analysis provides informative signals from a large number of probes, the analysis and interpretation of these signals bring with them certain inherent limitations. First, the complex dependency of signals on the probe sequences. It is well known, for example, that the hybridization energy of probes and their target nucleotides is dependent on the probe sequences to the extent that the signal intensities of probes are significantly different, even when the concentrations of the target nucleotide are identical [11], [12], [13]. Second, the existence of inappropriate signals because of non-specific binding between the probe and target, an inevitable phenomenon when a complex mixture of DNA or RNA fragments are hybridized to millions of probes simultaneously [14], [15]. Several methods have been proposed to overcome these difficulties and improve the accuracy and sensitivity of microarrays for expression analysis [16], [17], [18], [19]. For example, we previously proposed a thermodynamic model of hybridization that considered non-linear effects of probe-target interaction, in which the parameters representing the dependence of hybridization free energy on probe sequences were obtained by fitting between the expected and observed probe signals [20]. We also developed an algorithm to estimate non-specific binding between probe and target by using a similar thermodynamic model, with which non-specific signals can be predicted with high accuracy (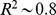) [22]. Using these thermodynamic models, we have been able to significantly improve the accuracy and dynamic range of mRNA quantification. However, so far, similar precise thermodynamic modeling has not been applied for the detection of sequence substitution, and the sequence dependence of signal intensities, including the effect of single or multiple mismatches between probes and target genomic DNA fragments, remains unclear.

In this study, we have developed a novel algorithm that uses an appropriate thermodynamic model of hybridization to detect single-base substitutions by using microarray data. Into this thermodynamic model, we introduced a penalty for mismatches, which represents the effects of substitutions on hybridization affinity. To evaluate the detection accuracy of sequence substitutions, we used a high-density tiling microarray designed for the *E. coli* W3110 strain. To detect differences in the genomic sequences between the different strains, we applied the genomic DNA of a previously sequenced *E. coli* strain DH1 ME8569 [21] to this tiling microarray, and successfully identified 219 out of 227 known single-base substitutions. This detection accuracy is significantly higher than that of other methods as SNPscanner, indicating that incorporation of hybridization free energy into analysis models can improve the detection of sequence variants by using microarray data.

## Methods

### Design of resequencing microarray

**Figure 1:**
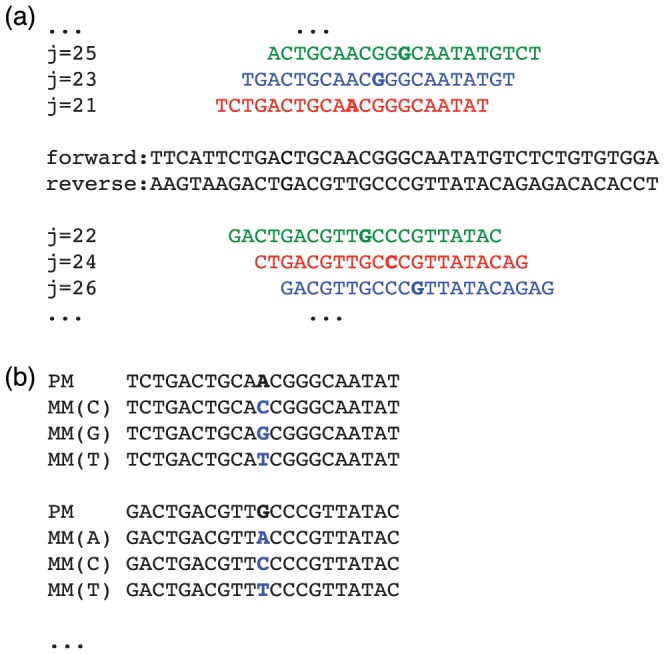
Schematic illustration of the design of the resequencing array. (a) Both forward and reverse strands were tiled alternatively, in 1 bp resolution. Probes were divided into 3 arrays cyclically (shown by red, green, and blue). (b) For each perfect match (PM) probe, the central base in the probe (shown by bold) is substituted. Three mismatch (MM) probes were designed by substituting the central base into other bases (shown by small letters).

A fine-tuned resequencing array covering the whole genome of E. coli W3110 strain was newly developed according to the Affymetrix CustomExpress Arrays. Figure 1 shows the design of the high-density tiling microarray. First, we designed tiling probes corresponding to the entire reference genome at 1 base pair (bp) resolution by using 21-bp-long perfect match (PM) probes. Probes corresponding to the forward and reverse strand were designed alternatively. In addition, for each PM probe, we designed all 3 possible mismatch (MM) probes, whose central base was substituted with other base types. Note that, we define “probe position” in the genome according to the position of the central (11th) base.

Because the genome size of *E. coli* is approximately 4.65 M bp, 18.6 million probes were required to cover the whole genome, in addition to all possible substitutions described above. All 18.6 million probes were represented on 3 Affymetrix CustomExpress arrays, each containing 5 million probes per array in 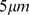 format.

### Thermodynamic model of hybridization

For the detection of nucleotide substitution, we developed a model to estimate the number of target molecules hybridized to probes based on a Finite Hybridization (FH) model [20], [22]. In this model, the intensity of a probe is estimated as a function of the probe sequence and the target concentrations. We let the sequence of the ith PM probe, whose central (11th) base is the ith base in the genome, be given by 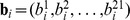, where 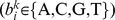 represents the base at k-th position of probe. Then, we assumed that the hybridization free energy 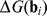 between the probe and the specific target, i.e., the target DNA fragment without mismatch to the corresponding PM probe, can be estimated by the expanded position-dependent nearest neighbor model proposed in [20] as follows:
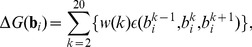
(1)where 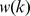 denotes a position dependent weight that decreases as it approaches the probe ends, and 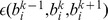 represents the local hybridization energy at kth position that depends on the types of the adjacent 3 base pairs.

Next, we considered the penalty in the hybridization free energy due to mismatches between the probe sequence and the target. Here, we assumed that when there is a single base mismatch at the 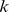-th position of the 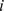
th probe, a penalty term 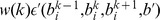 is simply subtracted from 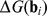, where 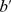 represents the substituted base type at the 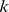-th position, and 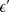 denotes the energy penalty. We estimated the decrease of hybridization efficiency in the MM probe by using 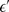 because the MM probes we designed contain a single mismatch between the probe and target, even when there is no base substitution in the sample genomic DNA. Moreover, when the sample genomic DNA contains a single-base substitution at a certain position, MM probes around this substitution have 2 mismatches relative to the target, and we estimated the hybridization free energy of such MM probes by subtracting 2 energy penalty terms corresponding to 2 mismatches from 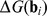. We further assumed that the number of base substitutions in the sample genomic DNA is sufficiently small to allow us to neglect cases in which multiple substitutions occur within a probe length (i.e., 21 bp).

We also estimated the hybridization free energy for the non-specific hybridization, 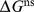, according to [22]. Here, this free energy was assumed to be represented by the expanded position-dependent nearest neighbor model, as follows:
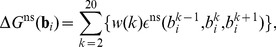
(2)where 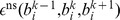 denotes the local hybridization energy for non-specific targets at the k-th position. Furthermore, following the results of our previous study [22], we also considered the effect of self-folding of probes by computing expected free energy of folding 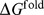 using UNAfold [23], which is based on the model proposed by Zuker and others [24], [25].

On the basis of the above free energy terms, the reaction coefficients for the probe-target hybridization, non-specific hybridization, and probe folding for the 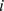
th probe were calculated as 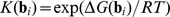, 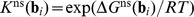, and 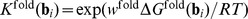, respectively, where 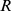 and 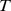 represent the gas constant and temperature, and 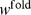 is a scaling constant as an adjustable parameter. Hence, by considering the finiteness of both probe and target molecules [20], [22], the log-transformed signal intensity of the 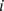
th probe is estimated as follows:
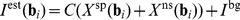
(3)

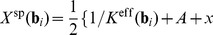
(4)

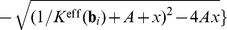


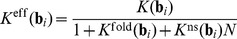
(5)

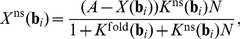
(6)where 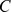 and 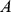 represent the scale coefficient and the total probe concentration, respectively, 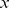 and 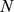 give the effective concentration of specific and non-specific targets assumed to be identical for all probes, and 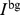 denotes the optical background intensity. In this model, 343 parameters are adjusted to fit the above expected intensities to the observed data: 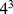 parameters for the hybridization free energy between specific target and PM probes 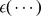; 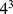 parameters for the hybridization free energy between non-specific target and probes 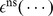; 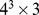 parameters for the energy penalty parameter 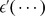; 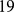 for the position dependence of the weight factors 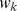; and 4 parameters for the scale constant 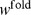, 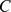, 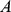 and the optical background constant 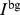.

### Parameter estimation

We determined the parameters in the above hybridization mode by fitting the estimated signal intensities of the probes to those observed experimentally. For the fitting, we obtained the intensity signals using the genomic DNA that was used to design the tiling microarray, and computed the residual sum of squares 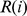 at the 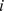
th position in the genome, as follows:
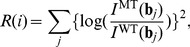
(7)

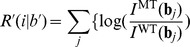


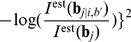
(8)where 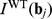 and 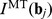 represent the observed intensity of the probe at the 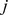
th position on the genome, for the wild-type and mutant strain, respectively. 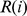 denotes the sum of squared log-intensity ratio of the mutant strain to the wild type, at the 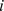-th position in the genome. 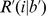 denotes the sum of squared error between the observed and estimated intensity ratio, where 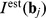 represents the estimated signal intensity for 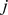
th probe, assuming no mismatch, while 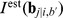 estimates the intensity assuming that the 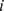
th base in the genome is substituted to a different base, 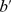. We then summed over all PM and MM probes containing the 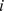
th base, i.e., the PM probes ranging 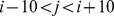 and corresponding MM probes. Note that, in case of MM probes, a mismatch should exist at the center of the probe, and in case of 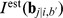, another mismatch exists because of mutation between probe and target sequence, and therefore, the energy penalty was subtracted from the hybridization free energy. The parameter values in the model were determined to minimize the sum of the above residual errors over all genome positions, by using a simple genetic algorithm implemented in R software [26].

### Detection of nucleotide substitution

To identify nucleotide substitutions in a genomic DNA sample that was compared to the reference sequence of *E. coli* W3110, we first calculated the above residual sum of squares 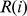 for all genome positions by using the parameter values determined by the above fitting procedure. In theory, when a single-base substitution exists at the 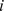
th genome position in comparison with the reference genome, the residual sum of squares 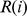 becomes significantly larger than those without substitution, and the residual sums be used to identify the position of the substitution. In practice, however, when a substitution exists at the 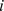
th position, residual errors around the substitution, i.e., 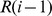, 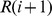, 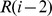, 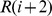, 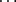, were increased. Therefore, for the initial screening of nucleotide substitutions in the genome sample, we identified a candidate region of the genome, in which the running average of residual errors 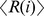 was larger than a given threshold value 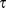. Here, we considered only regions equal to or longer than 10 bp and smaller than 80 bp. When regions smaller than 10 bp exhibited average large residual errors, this may be due to experimental errors, while in the case of longer regions, this is not due to single-base substitution, but due to a structural mutation, such as deletion. Such cases were neglected, because in this algorithm, we focused on the detection of single-base substitutions only.

After the initial screening to identify genomic regions that might contain nucleotide substitution, we further analyzed the residual errors between the estimated and observed signal intensity ratios to determine the precise position and base type of the substitution. Here, assuming that the relative error between the observed and estimated intensities follows a normal distribution with a constant variance, a log likelihood ratio 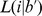 for testing the hypothesis that 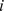-th base is the original base in the reference genome versus substitution to 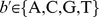 is given by,
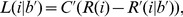
(9)where 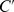 is a constant coefficient that can be neglected when we compare only relative differences of the value of 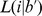. Because we assumed a normal distribution of the intensity ratio, the likelihood ratio is proportional to the difference between 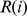 and 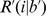. The value of 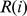 may increase because of some signal noise, but in such cases, the value of 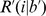 will not decrease. On the other hand, where a base in the genome was substituted to another base 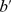 by mutation, the value of 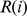 will increase, and that of 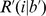 will decrease. A large positive value of 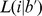 indicates a greater likelihood that the base type 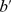 will be detected at the 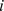
th position than the original base type in the reference genome. To determine the position and base type of the substitution, we sought the position 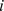 and base type 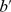 that maximize the likelihood ratio 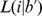 in the candidate region. Then, when the maximum likelihood ratio exceeds a given threshold 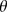, we called the base substitution occur to base type 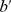 at 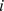
th position. In this detection algorithm, there are 2 parameters 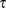 and 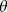 that change the sensitivity and specificity of the detection.

### Reference sequences

The reference genome sequences of *E. coli* W3110 and DH1 ME8569 strain were compared using MUMmer [27], [28] and 259 single base substitutions were identified. Substitutions meeting the following criteria were removed from further evaluation because these substitutions are difficult to detect using this microarray-based method: (1) another neighboring substitution occurs within 21 bp; (2) multiple copies of the sequence, i.e., the same sequence longer than 21 bp, exist in the genome; (3) the GC content of 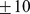 bp is either too low (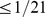) or too high (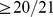), After removal of these conditions, we used the remaining 227 single-base substitutions as reference single-base substitutions.

### Data acquisition

E. coli W3110 and DH1 [21] were obtained from the National BioResource Project at the National Institute of Genetics, Shizuoka, Japan. Genomic DNA of these strains was isolated and purified using an Wizard Genomic DNA Purification Kit (Promega) in accordance with the manufacturer's instructions. For sample preparation of genomic DNA, standard methods for fragmentation and end-terminus biotin labeling were carried out following the Affymetrix protocols with slight modifications. Hybridization, washing, staining, and scanning were also carried out according to the Affymetrix protocols. Following washing and staining, the arrays were scanned using a Gene-Chip Scanner 3000 (Affymetrix). Absolute signal intensities of every probe in every sample were generated using GCOS 1.0 software (Affymetrix). The extracted microarray data were analyzed using custom-designed scripts in R software [26].

## Results and Discussion

### Detection of single-nucleotide substitutions by FH model

To evaluate the accuracy of the FH model for detection of single-nucleotide substitutions, we applied a method, in which we compared the intensity data obtained from the genomic DNA samples of *E. coli* strains DH1 and W3110 as the reference. In comparison with the reference genome, the genomic DNA of *E. coli* DH1 has 227 known single-base substitutions, which are detectable by this array-based approach. We first determined the 343 parameters in the FH model described in the Materials and Methods section by minimizing the average residual sum 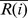 over the whole genome that was calculated from the intensity data of the reference genome. Because the tiling microarray we used contains large MM probes, we can estimate the mismatch energy penalty by fitting to the physical model of hybridization while considering the mismatch. After this parameter fitting using the reference genome, all parameter values were fixed and used throughout this study.

Next, by using the parameter values, we screened candidate genomic regions containing alterations by identifying regions showing higher values of the residual error 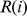 than a threshold 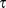. Here, we empirically set this parameter 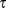 to 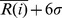, where 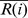 denotes the average of the residual error 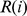 over the whole genome and 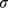 represents the standard deviation of 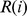. We identified 2 large regions showing significantly large residual errors (11 Kbp and 15 Kbp) and we confirmed that these regions corresponded to gene deletions fixed in DH1 genome sequence in comparison with the W3110 genome. After removing such gene deletions from the analysis, 4030 candidate regions showing large 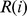 were left, which might correspond to single-nucleotide substitution.

**Figure 2:**
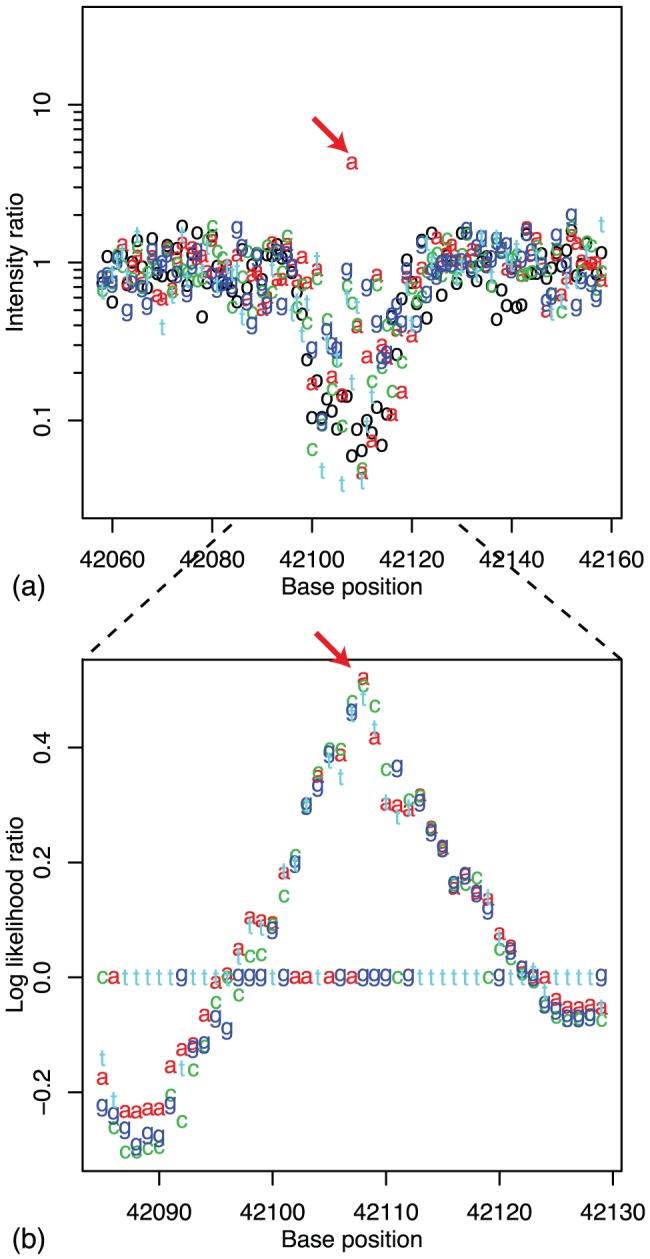
Detection of substitution. a) Signal intensity ratio of DH1 to W3110 in a candidate region. The horizontal axis represents the position of the probe in the genome and the vertical axis represents intensity ratio of the DH1 to the wild type. Black circles represent the intensity ratio of PM probes, and letters a,c,g,t represent those of MM probes with corresponding substitutions. The intensity in DH1 drops around a mutation due to mismatch, but there is a mismatch probe that shows higher intensity in DH1 than that in W3110 (show by the red arrow). (b) Log likelihood ratio evaluated from the intensity ratio. The vertical axis represents the likelihood that there is a mutation at that position.

**Figure 3:**
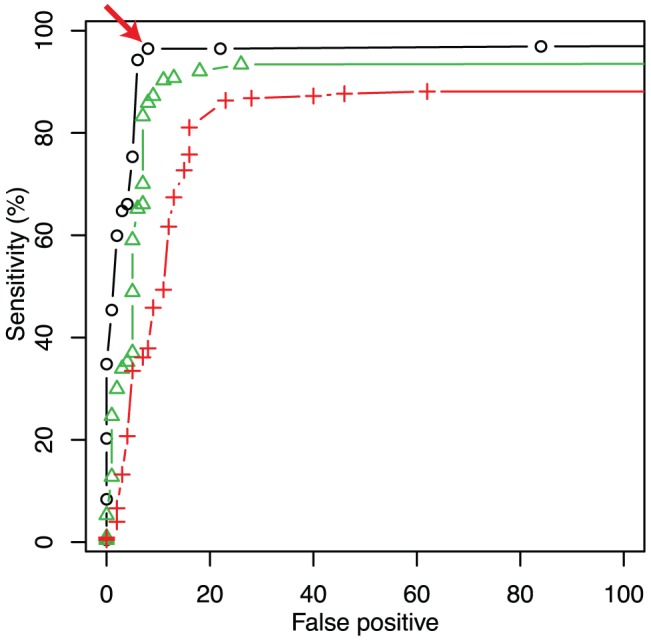
Comparison to SNPscanner. Black, green, and red lines represent sensitivity of our method, our method using a single array, and SNPscanner, respectively. The horizontal axis shows the number of false positive (FP) call, while the vertical axis represent the sensitivity sensitivity defined by the number of detected true positive (TP) divided by the total number of substitutions. The red arrow corresponds to the parameter by which TP-FP was maximized (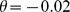), where 219 substations out of the known 227 ones were correctly detected, while 8 FPs were detected. As shown, the sensitivity of our method (black and green) is superior to SNPscanner (red) independently of the threshold values for the substitution detection.

To detect single-nucleotide substitutions, we next evaluated the likelihood of all possible substitutions within each candidate region. Figure 2(a) shows an example of the intensity ratio 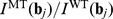 at a candidate region. In this genomic region, the raw signal intensities of the DH1 sample generally decreased in comparison with those of the W3110 sample, because of the single-base substitution at the position indicated by the arrow. On the other hand, at the position indicated by the arrow, the intensity of a single MM probe increased compared to that in the wild type, because the single-base substitution in the DH1 genome made this MM probe into a PM probe. Figure 2(b) represents the log likelihood ratio 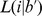 calculated from the data in Figure 2(a). Each character represents the central base 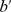 used for the calculation of the likelihood ratio. We sought the single-base substitution that maximizes this likelihood ratio in the candidate region, e.g., in the case of Figure 2(b), the substitution to base A at the position indicated by the arrow maximized the likelihood ratio, which might indicate that substitution to adenine occurred at this position. However, because this screening might still generate false-positive calls, we chose candidate substitutions that exhibit log likelihood ratios larger than a threshold value 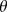 for the final call of the substitution detection. Because in this analysis, the true position and base type of all single-base substitutions fixed in the DH1 genome were known, we investigated the dependence of sensitivity, defined by the number of detected TP divided by the total number of substitutions, on the parameter 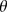. The black line in Figure 3 shows the change in sensitivity changes with 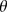, where the x-axis indicates the number of false positives (FP). The red arrow in this figure represents the data point that maximizes the number of true positives (TP) 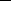 FP (with the threshold 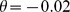). With this parameter value of 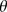, 227 substitutions were called by our algorithm. Of these substitutions, we confirmed that 219 were located within 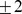 bases of the true substitutions, corresponding to the 8 FP calls. Moreover, because the total number of substitutions we analyzed between DH1 and W3110 genomes was 227, 8 substitutions were not identified (i.e., false negatives). Among 219 calls that were called close to the true substitutions, 213 were called at the exact position, while 212 were identified correctly with respect to both positions and base types. It should be noted that, of the 8 FP calls, 6 calls corresponded to the insertion of transposons in the DH1 genome.

### Comparison with SNPscanner

To evaluate the detection performance of our method, we compared the detection sensitivity and specificity with the widely used detection algorithm “SNPscanner” proposed by Gresham *et al.*, which is also based on likelihood estimation [7]. The essential difference between our method and SNPscanner is that the former considers the physical model of hybridization to represent intensity changes by mutations, while the latter is based on a linear regression model of intensity ratio between the control and the sample strains. It should be noted that SNPscanner can use only the data of MM probes whose central base is complementary to that of corresponding PM probes, and that other possible types of MM probe cannot be included in for this analysis. Accordingly, we removed intensity data of such non-complementary MM probes from both tiling microarray datasets for the comparison of these 2 methods. Moreover, we removed two-thirds of perfect and MM probes to meet the limit of the number of intensity data in SNPscanner. After removal of these probes, the resolution of the tiling array data became 3 bp, while the original data were at 1-bp resolution.

Figure 3 shows the sensitivities obtained by both methods. As shown, independently of the threshold values for the substitution detection, our detection algorithm exhibited higher sensitivity than SNPscanner, which is likely due to the incorporation of appropriate physical models to represent probe intensity in our method.

### Effect of tiling resolution on detection sensitivity

**Figure 4:**
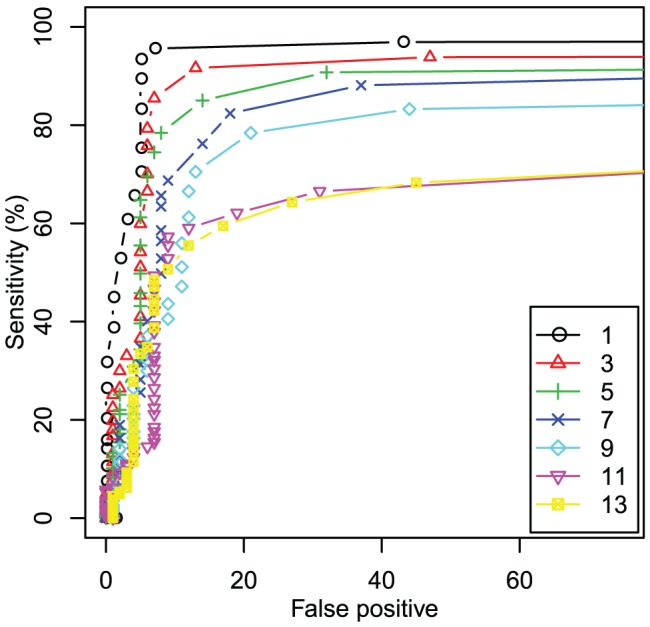
Detection sensitivity using reduced data sets. Lines represents the relationship between the number of false positive and the sensitivity as Fig. 3 with different probe resolutions. Since resolution is limited, we regard substitutions called within 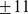 bp range of the true ones as correctly detected. As expected, the sensitivity decreases with the decrease of the probe resolution. For example, 1
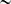
3 bp tiling resolution is necessary to achieve more than 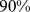 sensitivity.

Given the existence of various types of tiling microarrays with different probe resolutions, evaluation of the effect of probe resolution on the detection performance is important for probe design. Accordingly, using 1-bp-resolution data from our high-density tiling microarray, we investigated how the sensitivity of substitute detection depends on the probe resolution by removing probe data and reducing the tiling resolution to 3 bp, 5 bp, 7 bp, 9 bp, 11 bp, and 13 bp. In this analysis, we discarded non-complementary MM probes, and used the intensity data of PM and complementary MM probes only. Figure 4 shows the sensitivities obtained using various tiling resolutions and threshold parameters 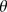. As anticipated, the detection sensitivity decreases with the decrease of tiling resolution, i.e., the number of probes covering each base position. For example, 209 (92%) substitutions were identified in the case of 3 bp resolution, where each base pair on the genome is covered with 7 different probes. This result suggested that, for the reliable identification of substitutions (e.g., 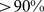 sensitivity), the tiling resolution should be 5 bp or less.

In conclusion, in this paper, we have presented a new method to identify single-base substitutions based on tiling microarray data by using a thermodynamic model of hybridization between probes and target DNA fragments. By introducing an energy penalty for hybridization free energy due to mismatches between the probe sequence and the target, our model allows the prediction of differences in signal intensities between PM probes and probes with single or double substitutions. Using this model, single-nucleotide substitutions can be identified by the maximum likelihood method. We evaluated this method and showed that it can detect 219 (96.5%) of known substitutions by comparing the microarray data of 2 different E. coli strains. This detection performance was significantly higher than that of an existing method, presumably because of the appropriate modeling of hybridization process. We also showed that the sensitivity of the detection depends on the tiling resolution of the microarray, and found that a resolution of 5 bp or less is necessary for accurate detection of single-base substitutions. It should be note that, since this model can predict absolute signal intensities based on the sequence of probes, we can detect the amplification of a specific region of genome from the change of signal intensities accurately. In this study, we use the genomic DNA of *E. coli* as an example to evaluate our methods for microarray-based SNP detection. The results depend only on the physical properties of genomic DNA and oligo nucleotide probes, while they should be independent of the sources of genomic DNA including prokaryotes and eukaryotes after appropriate extraction of genomic DNA. Thus, we expect that this method will be useful for the other model systems and genomes for future development of microarray-based SNP detection.

Due to the recent advances in Next-gen sequencer techniques, the use of microarray for SNP detection decreased its impact. However, still it has advantages on the simplicity of experimental procedure and low costs, and thus even though it is not used for advanced researches, it can be used for consumer application as medical device for diagnosis. Therefore, a method to overcome the disadvantages of this method, as low accuracy and high false positive rate, is desirable, and we expect that our study contributes the improvement of the microarray-based SNP detection. We anticipate that our method will improve the detection performance of single-nucleotide substitution in tiling microarray data.
